# Development and Evaluation of an Innovative Approach Using Niosomes Based Polymeric Microneedles to Deliver Dual Antioxidant Drugs

**DOI:** 10.3390/polym15081962

**Published:** 2023-04-20

**Authors:** Ahlam Zaid Alkilani, Hadeel Abo-Zour, Haneen A. Basheer, Hana Abu-Zour, Ryan F. Donnelly

**Affiliations:** 1 Department of Pharmacy, Faculty of Pharmacy, Zarqa University, Zarqa 13110, Jordan; 2 Medical Biology Centre, School of Pharmacy, Queen’s University Belfast, Belfast BT7 1NN, UK

**Keywords:** ascorbic acid, caffeine, permeation, polymers, microneedles, niosomes, antioxidant

## Abstract

Ascorbic acid (AA) and caffeine (CAFF) work to protect cells from ultraviolet (UV) radiation and slow down the photoaging process of the skin. However, cosmetic application of AA and CAFF is limited due to poor penetration across the skin and rapid oxidation of AA. The aim of this study was to design and evaluate the dermal delivery of dual antioxidants utilizing microneedles (MNs) loaded with AA and CAFF niosomes. The niosomal nanovesicles were prepared using the thin film method and had particle sizes ranging from 130.6–411.2 nm and a negative Zeta potential of around −35 mV. The niosomal formulation was then combined with polyvinylpyrrolidone (PVP) and polyethylene glycol 400 (PEG 400) to create an aqueous polymer solution. The best skin deposition of AA and CAFF was achieved with the formulation containing 5% PEG 400 (M3) and PVP. Furthermore, the role of AA and CAFF as antioxidants in preventing cancer formation has been well-established. Here we validated the antioxidant properties of ascorbic acid (AA) and caffeine (CAFF) in a novel niosomal formulation referred to as M3 by testing its ability to prevent H_2_O_2_-indued cell damage and apoptosis in MCF-7 breast cancer cells. Results showed that M3 was able to shield MCF-7 cells from H_2_O_2_ induced damage at concentrations below 2.1 µg/mL for AA and 1.05 µg/mL for CAFF, and also exhibited anticancer effects at higher concentrations of 210 µg/mL for AA and 105 µg/mL. The formulations were stable for two months at room temperature in terms of moisture and drug content. The use of MNs and niosomal carriers could be a promising approach for dermal delivery of hydrophilic drugs like AA and CAFF.

## 1. Introduction

The skin acts as an efficient barrier between the body and its surroundings, preventing harmful chemicals from entering the body [1]. Furthermore, this barrier prevents hydrophilic and large molecular weight compounds (>500 Da) from penetrating the intact skin [1,2]. Various chemical and physical techniques have been developed to improve the transport of a wide range of drugs over the skin, including the use of chemical enhancers, iontophoresis, microneedles (MNs), and ultrasound [3,4]. Over the last few decades, nanocarrier-based drug delivery has made considerable advances. The selection of the nanocarrier system is based on the physicochemical characteristics of the drug [5,6]. Recently, niosome research has gained a lot of interest as a nanocarrier system because of its numerous uses, such as ability to encapsulate lipophilic or hydrophilic drug, and being incorporated into a variety of drug administration routes [7,8,9,10]. Niosomes are surfactant-based vesicles that have advantages over liposomes in terms of improved stability, smaller particle sizes, and lower cost [11,12]. They have been used in the cosmetic industry for dermatological purposes because of their ability to increase skin penetration and the stability of entrapped drugs [13]. Additionally, physical methods, such as MNs, can be utilized to improve the penetration of active molecules into the skin through epidermal perforation [9]. MNs are distinguished by its low invasiveness, and self-administration, which can result in good patient compliance as well as reduced hazardous waste sharps1 [14,15]. The aforementioned characteristics indicate a significant interest in microneedling applications within the drug delivery and cosmetics industries. In dissolving MNs (DMN), the nanocarrier can be loaded within a biodegradable matrix that is dissolved when put into the skin without producing any biohazardous waste [16,17]. Polyvinylpyrrolidone (PVP) and polyethylene glycol (PEG) are widely used polymers in drug delivery applications due to their biocompatibility and ability to enhance drug solubility and permeability [18,19]. Various studies have utilized PVP and PEG-based formulations for drug delivery using different routes of administration such as oral, and transdermal [20,21]. For example, in a study carried out by Homayouni et al. [22], it was discovered that PVP had a more significant impact on enhancing the dissolution rate compared to other carriers. PVP, which has a higher Tg and greater effect on dissolution rate, may be a more suitable carrier than polymers for solid dispersion formulation of poorly water-soluble drug, according to the findings of the study. In the context of microneedles, PVP and PEG have also been explored as materials for microneedle fabrication due to their ability to provide mechanical strength and facilitate drug release. Yang et al. [21] described the development of a microneedle patch made from PVP. Gamma rays were used to prepare the patch, and the back side of the patch was coated with either gold or silver using a method called thermal evaporation. This coating served as bioelectrodes to increase and regulate the drug release efficiency. The microneedle patch coated with Au or Ag was strong enough to puncture the skin successfully. The researchers confirmed that by applying thermal or electrical stimulus, the drug release from a drug-loaded microneedle patch was increased, as demonstrated by tests using a Franz cell.

Reactive oxygen species (ROS) cause oxidative stress, which accelerates the aging process of the skin and development of cancer [23]. Oxidative stress also causes chronic inflammation, which can lead to collagen fragmentation, the reorganization of collagen fibers, and exacerbation of skin pigmentation [24,25]. More than 80% of environmental ROS that damage the skin is produced by UV radiation [26]. Antioxidants are a useful strategy for preventing the signs of photo-induced skin aging. Ascorbic acid (AA) is found in high concentrations in normal and healthy skin, where it supports a number of functions, including promoting collagen formation and contributing in antioxidant defense against UV-induced photodamage. The epidermis has higher AA levels than the dermis [27]. However, AA levels have been found to be lower in naturally aged or photodamaged skin, according to several studies [27,28,29]. In addition, caffeine (CAFF) limits photodamage, decreases skin roughness and wrinkle formation [30]. It has antioxidant properties which helps in protecting cells against the UV radiation and slows down the process of photoaging [31]. The use of CAFF in cosmetic products with concentrations up to 3% is considered safe, and not toxic to human skin [32]. While some researchers loaded AA and CAFF onto niosomes [33,34], others prepared them as a MN array [35,36]. However, none of these studies involved loading AA and CAFF as niosomes onto the MN array. Dangol and colleagues [35] reported that they were able to successfully load caffeine onto dissolving microneedles by inhibiting its crystal growth using hyaluronic acid, which served as the matrix material for the dissolving microneedle. Additionally, the researchers evaluated the anti-obesity activity of caffeine in mice with diet-induced obesity. After administering the caffeine-loaded dissolving microneedle patch for 6 weeks, there was a significant improvement in lipolysis as indicated by the activity of leptin and adiponectin, which resulted in significant weight loss. In addition, Sawutdeechaikul et al. [36] conducted a study in which they used a special dissolvable microneedle called the detachable dissolvable microneedle array to embed a model compound into both the epidermis and dermis. The MNs allows for the needle to detach from the base within 2 min after administration, and the researchers demonstrated the diffusion of the compound to surrounding skin tissue at various post-administration time points. By co-loading glutathione with AA, the researchers were able to stabilize AA.

The limited penetration of AA and CAFF through the skin is due to the hydrophobic nature of the *Stratum corneum* and their water-soluble properties (log p −1.6 and −0.07 respectively). AA is also challenging to include in pharmaceutical preparations due to its instability issues, as it is susceptible to oxidative degradation from air and light exposure. This weak skin penetration limits the cosmetic application of AA and CAFF. To tackle this issue, a new dermal delivery method has been devised using nanocarriers and microneedles (MNs) to enhance drug stability and enhance skin penetration. The goal of this study is to use niosomes as a drug carrier to deliver AA and CAFF to the skin through MNs, forming a nanocarrier-based system and to evaluate such formulation for cancer treatment.

## 2. Materials and Methods

AA and CAFF were purchased from MEDEX™ (Rugby, UK), acetic acid (HPLC grade), acetonitrile (HPLC grade), chloroform (HPLC grade) and methanol(HPLC grade) were purchased from Tedia™ (Fairfield, OH, USA). Span™ 60, Tween™ 60, Tween™ 80, phosphate buffered saline (PBS) tablets and cholesterol (Chol) were purchased from Sigma Aldrich™ (Dorset, UK). Methylene blue and polyethylene glycol 400 were provided from GCC Diagnostics™ (Flintshire, UK). Dihexadecyl phosphate (DCP), and polyvinyl pyrrolidone (molecular weight 40,000 Da) were purchased from Sigma Aldrich™ (Dorset, UK). Cellulose dialysis membranes with a molecular weight cut-off (MWCO) of 12–14 kDa was purchased from Himedia Laboratories™ (Maharashtra, India). RPMI 1640 medium with L-Glutamine was purchased from Caisson Labs (Smithfield, UT, USA). Penicillin–Streptomycin Solution 100× was provided from Euroclone™ (Milan, Italy). Cytiva™ HyClone Fetal Bovine Serum was purchased from Global Life Sciences™ (Pasching, Austria). The breast MCF-7 cell line was obtained from Dr. Ahmad Sharab of the University of Jordan [37]. All other chemicals used in this study were of analytical grade.

### 2.1. Methods

#### Preparation of Niosomes

**Figure 1:**
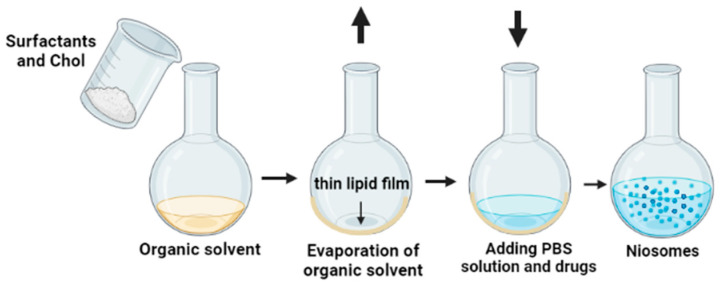
Schematic representation of the formulation of AA and CAFF niosomal nanoparticle.

AA and CAFF loaded niosomes were prepared via thin film hydration (TFH). Briefly, niosomes of AA and CAFF were pre-pared by dissolving nonionic surfactants (Span 60, Tween 60, and Tween 80), cholesterol and DCP in 10mL organic mixture of (chloroform/methanol, 4:1, *v*/*v*) in a round-bottom flask. The organic mixture was evaporated under reduced pressure at 60 °C, and 120 rpm. The resulting thin lipid films was hydrated with 10 mL of a PBS solution that contained AA and CAFF at 60 °C for one h as shown in Figure 1. All niosome dispersions were stored in a refrigerator at 4 °C. The composition of different niosomal formulations is shown in Table 1.

### 2.2. Characterization of Niosomal Nanoparticles

#### 2.2.1. Transmission Electron Microscope (TEM)

The morphology of the niosomes was examined using a transmission electron microscope (TEM, FEI Morgani 268, operating voltage of 60 kV, Eindhoven, The Netherlands) and Mega View II digital camera. Niosomes were dispersed on a copper grid coated with carbon and diluted with distilled water prior to imaging. To analyze the morphology of the niosomes, Image J was employed.

#### 2.2.2. Particle Size (PS) and Zeta Potential (ZP)

To evaluate the PS and polydispersity index (PDI) of the chosen formula, dynamic light scattering (DLS) was employed using a particle size analyzer (Brookhaven 90 plus, Holtsville, NY, USA). Additionally, ZP of the particles was measured using electrophoretic light scattering (ELS) technique. To prepare the sample, 0.5 mg of niosomes were dispersed in 2 mL of distilled water at 25 °C. All experiments were carried out three times, and the results were presented as mean ± standard deviation (SD).

#### 2.2.3. Drug Entrapment Efficiency

The encapsulation efficiency (EE%) of all the niosomal formulations was assessed using a Beckman Optima LE-80 K Ultracentrifuge. Briefly, a niosomal suspension was poured into 1.5 mL Eppendorf tubes and then subjected to centrifugation for 1 h at 16,000× *g* and 4 °C in a refrigerated centrifuge. Subsequently, the supernatant was discarded, and the separated niosomes were washed with PBS and then centrifuged again for two more cycles under the same conditions. To determine the amount of drug entrapped, 1 mL of isopropanol was added to 0.1 mL of the separated niosomes. The resulting mixture was then diluted with PBS up to 20 mL, sonicated, and then subjected to centrifugation again at 14,000× *g* for 15 min at 25 °C to obtain a clear solution that could be used for HPLC analysis.

EE% = (Amount of drug entrapped)/(Total amount) × 100%(1)

The “total amount” refers to the entire amount of either AA or CAFF that was used in the preparation process. Meanwhile, the “amount of drug entrapped” refers to the actual amount of drug molecules that have been successfully encapsulated within the vesicles.

### 2.3. Attenuated Total Reflectance—Fourier Transform Infrared Spectroscopy (ATR-FTIR)

The Perkin Elmer UATR-II device (Waltham, MA, USA) was utilized to investigate potential compatibility issues using ATR-FTIR spectroscopy. The samples were analyzed in the wavenumber range of 4000–400 cm^−1^ with a resolution of 2 cm^−1^ and 32 scans taken per sample. The collected spectral data was saved in. CSV format and analyzed with Ira Version 1 [38]. The ATR-FTIR method was used to evaluate the spectra of AA, CAFF, Span 60, Chol and Tween 80.

### 2.4. Stability Studies

A short-term stability study was conducted to assess changes in color, PS, PDI, ZP, and EE% over a two-month period. Three niosomal formulations (S4, S7, and S9) were chosen for the study based on the results of their characterization. The selected formulations were kept at 4 °C during the study.

### 2.5. In Vitro Drug Release Study

To conduct an in vitro release study, the dialysis method was utilized. The cellulose dialysis membrane, which had a molecular weight cutoff (MWCO) of 12–14 kDa, was immersed in PBS (pH 7.4) for 24 h. The release of AA and CAFF from the S4 and S7 formulations was evaluated using Franz-diffusion cells with a receptor volume of 12 mL (PremeGear, Hellertown, PA, USA). 0.5 mL of the niosomal suspension was placed in a dialysis bag and placed in the PBS (pH 7.4) release media. The receptor media was agitated at 37 °C and 600 rpm using a magnetic bar throughout the experiment. Samples of 1 mL were collected at 0.25, 0.5, 1, 2, 3, 4, and 5 h and the same volume of fresh PBS was added after each collection. The samples were analyzed using HPLC to quantify the levels of AA and CAFF after dilution. This experiment was repeated three times.

### 2.6. Fabrication of Dissolving MN

**Figure 2:**
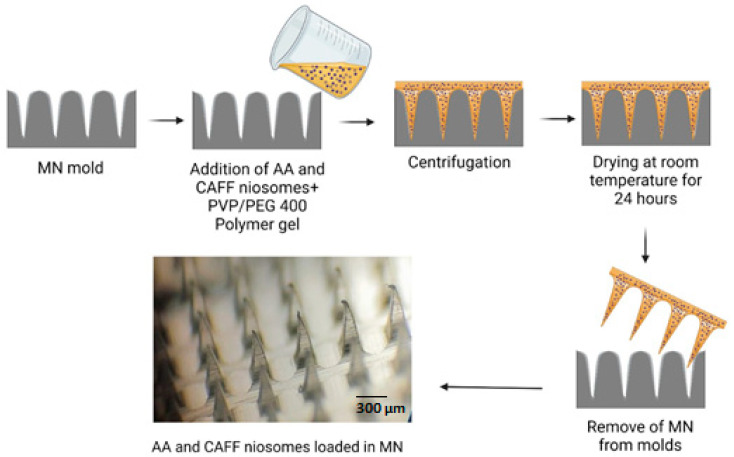
Schematic representation of the fabrication of MN loaded AA and CAFF niosomal nanoparticles.

Different biocompatible polymers, including polyvinyl alcohol (PVA) and polyvinylpyrrolidone (PVP), were utilized in different concentrations to make MNs formulations. To begin, an aqueous solution of the selected polymers was created; 60% (*w*/*v*) PVP. The solution was then combined with 0.3 mL of AA and CAFF niosomes and PEG 400, as summarized in Table 2. The mixture was stirred briefly to ensure the homogeneity of the niosomes before the MN casting process. The dispersion was poured into silicone molds to make 225 pyramidal needles with dimensions of 600 µm in height, 300 µm in width, and 300 µm in interspacing. The molds were then tightly sealed and centrifuged at 2000× *g* for 10 min. After that, the MNs were dried at room temperature for 24 h, as shown in Figure 2. The MNs were carefully removed from the molds and inspected visually using an optical microscope. Methylene blue was utilized as the model pharmaceutical and added to the matrix solution for characterization and evaluation.

### 2.7. Characterization of Dissolving MNs

#### 2.7.1. MNs Dissolution Rate

The in vitro dissolution profile of DMN was evaluated by inserting the prepared MNs into full thickness rat skin, for 1 min using manual application. The skin samples were prepared by shaving and soaking them in PBS (pH 7.4) for 15 min [39]. To prevent drying, the full thickness rat skin was placed on a wet tissue paper with PBS, with the dermal side facing down. MNs containing methylene blue were then manually applied into the skin for one minute before being removed at 5-, 10-, and 20-min intervals. The dissolution pattern of the MNs was then analyzed under a microscope.

#### 2.7.2. Evaluation of Skin Insertion Ability of DMN

A commercial polymeric film called Parafilm™ was utilized as a skin simulant for MN insertion research. This method was validated by Larrañeta et al. [40]. The Parafilm™ was folded into an eight-layer film with a thickness of 1 mm. The AA and CAFF niosome-loaded DMN were then inserted using manual pressure applied to the MNs baseplate for 1 min. After insertion, the MNs were removed from the Parafilm™ sheet. The sheet was then unfolded, and the penetrated needle holes were observed using an optical microscope. The insertion percentage for MNs was calculated by dividing the number of needle holes in each layer by the total number of needles [41].

#### 2.7.3. Determination of AA and CAFF Recovery from DMN

To find out the percentage of AA and CAFF recovered from M1, M2, M3, and M4, the samples were dissolved in 25 mL of the mobile phase containing 10% n-propanol using a sonicator for 10 min at 37 °C. The analysis was then performed using HPLC.

#### 2.7.4. Mechanical Characterization

To assess the height reduction of DMN, weights of 200 g, 500 g, and 1000 g were placed on the tips of the MNs for 5 min and then removed [42]. The shape and breakage of the DMN were then evaluated using an optical microscope, which indicates the mechanical strength.

### 2.8. Short Term Stability Study

M1, M2, M3, and M4 were accurately weighed and kept in containers with anhydrous calcium chloride to maintain dryness. After a period of two months, the appearance and organoleptic qualities (color and clarity) of the samples were examined. The moisture level and the content of AA and CAFF in the MNs were also determined.

### 2.9. Drug Permeation Study

In order to evaluate the permeation of AA and CAFF from DMN; M1, M2, M3, M4, S4 and S7 niosomes, we carried out ex vivo permeation studies using Franz diffusion cells (PremeGear, Hellertown, PA, USA) with an aperture diameter of 15 mm and a diffusion surface area of 1.76 cm^2^. Full thickness rat skin was taken from the back of a rat. The protocols rat skin preparation was approved by the Ethics Committee for scientific research; approval number (4/2335/27) dated September 2022. The rat skin was prepared and clamped between the donor and receptor compartments and the stratum corneum side was in contact with the donor phase. In the donor compartment, 0.5 mL of S4 and S7 niosomes as control. M1, M2, M3, and M4were manually pressed into rat skin for one minute. The SC side was connected to the donor phase and then the donor and receiver chambers were securely fastened using a clamp. To prevent evaporation, the area between the two chambers was covered with waterproof film (ParafilmTM). Following that, a 1 mL sample was taken from the receptor cell at different intervals of (0.25, 0.5, 1, 2, 3, 4, and 5) h. After each collection, the same volume of PBS solution was used to replace the samples. The samples were withdrawn and analyzed using HPLC. The cumulative amount of AA and CAFF permeated through rat skin per unit area (Q/A) was plotted versus time (t).

### 2.10. In Vitro Skin Deposition Analysis

To evaluate the presence of AA and CAFF in rat skin tissue, the skin was rinsed three times with PBS to remove any residual AA and CAFF. The skin was then diced, and AA and CAFF were extracted using methanol and sonication for 20 min. The skin was homogenized and centrifuged for 30 min at 12,000× *g*, and the resulting supernatant was further centrifuged for 30 min at 8000× *g*. The supernatant was then diluted for analysis using HPLC.

### 2.11. Kinetic of Drug Release

The Korsmeyer-Peppas and first order equations were fitted to the drug release rates for M3 formulation using LabPlot version 2.0 which are represented by the Equations (2) and (3) [43],

Mt = M∞ × (1 − e − kt)(2)

kt^n^ = Mt/M∞ (3)

where 

Mt: the amount of drug released at time (t).

M∞: the amount of drug released as t approaches infinity.

n: the diffusional exponent or drug release exponent.

K: the Korsmeyer release rate constant.

The function of selecting the best fit line was then given to the software. Suitable fit curves were only those with R^2^ ≥ 0.95 and sum of squared residuals (SSD) ≤ sum of squares.

### 2.12. Antioxidant Activity against H_2_O_2_ Induced Cytotoxicity

The antioxidant activity of M3 was tested against hydrogen peroxide (H_2_O_2_) induced cytotoxicity in MCF-7 cells. Cells were plated in 96-well plates at a density of 5000 cells/well, in the following day cells were or were not treated with M3 different serial dilutions of (21 + 10.5), (2.1 + 1.05), (0.21 + 0.105 and 0.0021 + 0.001) μg/mL) for 48 h, and then exposed to 800 µM H_2_O_2_, except for the control, for an additional 6 h to establish oxidative stress, Cell viability was then evaluated using the MTT assay as described earlier [44] after 96 h from seeding and the absorbance was measured using a microplate reader (Glomax™, Madison, WI, USA) at 560 and 750 nm. The percentage of cell survival versus drug concentration was plotted using Excel spreadsheets.

### 2.13. Cytotoxicity Assay

The MTT assay was used to determine the cytotoxicity of the M3 and blank M3 formulations using the MTT solution. For the toxicity studies, the cells were seeded in 96-well plates at a plating density of 5000 cells/well and incubated for 24 h. On the following day, different serial dilutions of the M3 formula ((210 + 105), (21 + 10.5), (2.1 + 1.05), (0.21 + 0.105), (0.021, 0.010) μg/mL) and for the blank M3 formula at serial dilutions ((180.2, 18.02, 1.8, 0.18, 0.018) mg/mL) were added to the appropriate wells and incubated for 72 h at 37 °C. After treatment, 0.02 mL of a 5 mg/mL of MTT solution (Sigma-Aldrich Inc., St. Louis, MO, USA) was added to each well and incubated for 4 h at 37 °C as described earlier [44]. Briefly, the MTT-containing media was then discarded, and the resulting violet formzan crystals were then solubilized by the addition of dimethylsulfoxide (DMSO; Sigma-Aldrich Inc., St. Louis, MO, USA). To assess the absorbance of the samples, the plates were analyzed using a microplate reader (Glomax™, Madison, WI, USA) at 560 and 750 nm. The percentage of cell survival versus drug concentration was plotted using Excel spreadsheets.

### 2.14. Analysis of the AA and CAFF

AA and CAFF were analyzed using a C8 column (300 mm × 0.45 mm; 5 μm, Phenomenex, Torrance, CA, USA) with a mobile phase consisting of 1% acetic acid mixed with acetonitrile (90:10 *v*/*v*%). The samples were filtered through 0.22 μm Minisart RC4 filters prior to analysis. The analysis was conducted using reverse-phase high-performance liquid chromatography (RP-HPLC) with a flow rate of 0.7 mL/min and an oven temperature of 40 °C. The HPLC system was (Shimadzu LC-20AT Pump, Standard Autosampler, SPD-20A UV/VIS Detector, Shimadzu, Kyoto, Japan). All injections for HPLC were 10 μL, and standard solutions for AA and CAFF were created by diluting stock solutions to a final concentration range of 10–190 μg/mL for AA and 5–95 μg/mL for CAFF. The detection of the drugs was performed at 286 nm.

### 2.15. Statistical Analysis

The statistical analysis of the results was carried out using either a *t*-test or a one-way analysis of variance (ANOVA). A statistically significant difference was determined as (*p* < 0.05) in all cases. GraphPad Prism software (version 6; GraphPad, Inc., San Diego, CA, USA) was utilized for this purpose.

## 3. Results and Discussion

### 3.1. Characterization of AA and CAFF Loaded Niosomes

**Figure 3:**
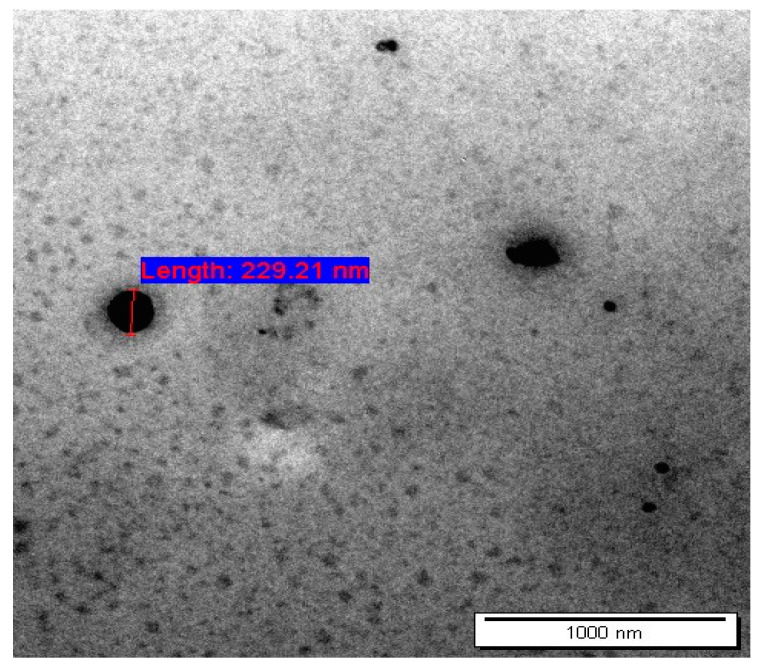
Transmission electron microscopy (TEM) micrographs of AA and CAFF-loaded niosome at a magnification of 50 kx.

Dermal delivery system (DDS) has several limitations owing to the skin’s barrier characteristics, which restrict drug penetration of hydrophilic drugs [45]. Niosomes extensively studied as an efficient dermal drug delivery since it enhances the permeation of the drug through skin and deposition in the dermis layer [46], when compared to other colloidal vesicle carriers, niosomes are more stable and cause less irritation [46]. In this study, niosomes have been formulated and evaluated as dermal delivery carriers for AA and CAFF. Nine formulations of AA and CAFF loaded-niosomes were successfully prepared using thin film method (TFH) as shown in Table 3. Niosomes were visualized by using TEM microscope, the vesicles had spherical shapes and were free of aggregation as shown in Figure 3. The PDI of the prepared niosome ranged from 0.005 to 0.256 indicating that the prepared niosomes were relatively homogeneous, since a formula that had a PDI value less than 0.3, demonstrated the homogeneity and monodispersed system [47,48]. Niosomal formulations were described as being milky white in color, odorless dispersions with a fluid-like consistency [49].

Table 3 summarizes the mean PS, PDI, and ZP of AA and CAFF-loaded niosomes. The PS of all the niosome formulations ranged from 130.6 ± 7.5 to 411.2 ± 4.51 nm. The size of niosomes is influenced by the HLB of the surfactant. The smallest size was seen in niosomes made with Span 60 without Tween 60 or Tween 80: 150.7 ± 10.1 for S1, 170.0 ± 3.7 for S2, and 130.6 ± 7.5 for S3. The size of niosomes increased with an increase in the HLB values of the surfactant. This is consistent with previous studies which showed that niosomes made of Span 60 and Brij 72 with lower HLB values (about 4.8) had smaller particle sizes compared to niosomes made of Tween 60 (HLB = 14.9) at the same cholesterol content [50]. The addition of Tween 60 and Tween 80 to the formulation significantly increased the size of niosomes (*p* value < 0.005), which may be due to the greater hydrophilic region of the Tween 60 molecule compared to Span 60.

One of the parameters of colloidal systems that is used to interpret their stability is their ZP [51]. Systems with the ZP value higher than ±30 mV are considered to be stable [52]. The composition of the niosomal bilayer and the amount and type of the charge-inducing agents affected the ZP of niosomes [51]. The ZP of niosomes ranged from 25.40 ± 0.52 to 50.2 ± 3.74, which is enough to ensure repulsion between the vesicles and prevent aggregation, resulting in stable niosomes [53].

All niosomes effectively incorporated AA and CAFF, and any untrapped drugs were removed through ultracentrifugation. Based on Table 3, the highest %EE of AA and CAFF from S7 formulation were 70.89 ± 0.42%, 67.07 ± 0.30%, respectively. This is in agreement with Aboubakr et al. who reported that the maximum %EE at 65 ± 3.6% by using cholesterol, Span 60 and Tween 80 [54]. Span 60 strongly interacts with cholesterol molecules and forms large core space for hydrophilic drug entrapments [54]. In addition, the presence of Tween 80 in the formulation enhanced the encapsulation of hydrophilic drugs, into the aqueous core of the niosomes [54]. Research has shown that no skin deposition occurs when the particle size of nanocarriers is larger than 600 nm [55], but carriers with a smaller particle size, such as 300 nm, can improve dermal delivery [56]. S4 and S7 were selected for further study due to having the highest drug entrapment efficiencies.

### 3.2. Attenuated Total Reflectance—Fourier Transform Infrared (ATR—FTIR) Analysis

**Figure 4:**
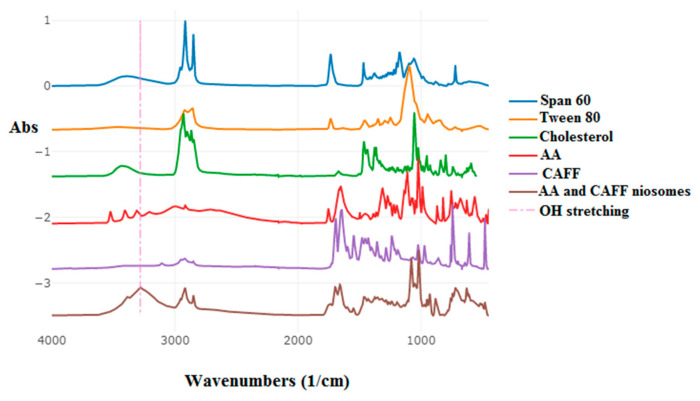
The FTIR spectrum of AA and CAFF niosome, Tween 80, (Span 60, and AA, CAFF and Chol.

The compatibility of AA and CAFF with the ingredients used to make the niosomal formulations was studied using ATR-FTIR. The IR spectra showed the main absorption bands for AA were at 1652 cm^−1^ (representing C=C stretching), 1753 cm^−1^ (due to stretching of the C=O in the lactone ring system), and 1313 cm^−1^ (representing the peak of enol-hydroxyl) [57]. For CAFF, the major peaks were at 1654 and 1644 cm^−1^, which were due to stretching of the C=O in cyclic hydrocarbons and the C=N in cyclic hydrocarbons, respectively [58]. The major peaks of Span 60 were at 3310 cm^−1^ and 2926 cm^−1^, indicating amines or the hydroxyl group and carboxylic acid, respectively. The spectrum of Tween 60 had a characteristic sharp peak around 1735 cm^−1^ which is attributed to the stretching vibration of ester carbonyl. Tween 80 shows many absorption peaks that are due to the different functional groups present in the molecules such as methyl group (−CH_3_) which shows absorption band around 2921 cm^−1^, while the band around 2854 cm^−1^ is due to −CH_2_-stretching [59]. The band around 1735 cm^−1^ can be attributed to C=O and the band at 1095 cm^−1^ is due to stretching of C–O–C, cholesterol shows the characteristics peaks at 3432 cm^−1^, 2930 cm^−1^ 1454 cm^−1^ and 1054 cm^−1^ due to stretching of the O-H, C-H, C=C, and bending of C-O [60]. The results showed that there was no interaction between AA, CAFF, and the excipients, as indicated by the unchanged major absorption bands of the characteristic peaks of AA and CAFF as shown in Figure 4. The wave number 3274 cm^−1^ in AA and CAFF niosome is attributed to hydrogen bonding between formulation components, which is known to be caused by interactions between the glycerol oxygen in Span 60 and the β-OH group in cholesterol, according to previous studies [61,62].

### 3.3. Stability Studies

The stability of niosome suspensions is a crucial aspect in considering these vesicles as a viable alternative to traditional delivery methods. Thus, the short-term stability of the niosomal formulations with the highest drug entrapment efficiencies, S4 and S7, was evaluated over a period of two months. The changes in color, PS, PDI, ZP, and %EE of the niosomes at a temperature of 4 °C over a two-month period are presented in Table 4. For a niosomal formulation to be considered stable, it must maintain consistent values of PS, PDI, ZP, %EE, and not experience phase separation or changes in color during storage.

The physical appearance and color of the stored niosomes did not alter, and there was no aggregation or precipitations signs observed after two months. As can be shown in Table 4, the PS of the niosomes did not change significantly (*p* > 0.05) at 4 °C. The %EE of AA from freshly prepared niosomes and after two months for S4 was 59.71 ± 0.09, and 59.46 ± 0.44, respectively. While %EE of CAFF from freshly prepared niosomes and after two months for S7 was70.89 ± 0.42, and 66.96 ± 0.14, respectively, indicating no significant changes (*p* > 0.05) was observed. All ZP values were within −35 mV throughout storage, indicating high formulation stability over storage time. The values of PDI were found to be between 0.02 and 0.25, which indicated the homogenous nature of the vesicles distribution and the niosomes stability [54]. The above results revealed that stable niosomes with appropriate particle size and excellent drug %EE were successfully produced.

### 3.4. In Vitro Release Study

**Figure 5:**
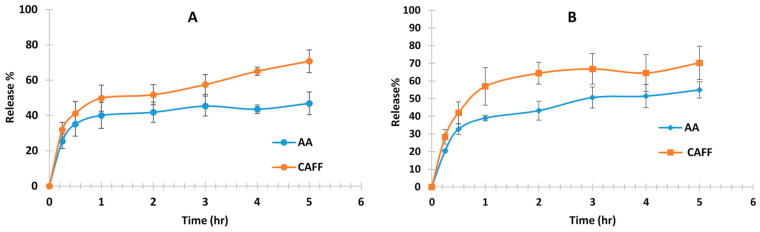
In vitro release studies of AA and CAFF from (**A**) S4 and (**B**) S7 niosome (n = 4).

The selected niosomal formulations, S4 and S7, which had the highest drug entrapment efficiency and stability, were chosen for in vitro release testing. The release patterns of AA and CAFF from these niosomal formulations are depicted in Figure 5. The results showed a fast release of the drugs from the niosomes. The initial rapid phase in the release profile may be due to the desorption of drugs from the surface of the niosomes while the slower phase observed in the last 4 h may be attributed to the diffusion of drugs from the core of the niosomes into the receiving medium through the bilayer [47], such as caffeine [34] and insulin [63] which highlights the potential of niosomal technology for the efficient and controlled delivery of hydrophilic drugs.

Release percentage of CAFF from S4 and S7 were 70.26 ± 9.45%, 70.71 ± 4.98%, respectively in 5 h. There is no significant difference in the release of CAFF between S4 and S7 (*p* value = 0.987). However, there is a significant difference (*p* = 0.014) in the release of AA between S4 and S7 since the release% of AA from S4 and S7 was 54.94 ± 4.57%, 46.83 ± 6.43%, respectively. Similar results were achieved in the in vitro release study of AA and α-tocopherolfrom niosomes, similar biphasic release was observed [64]. The rapid initial phase may be related to desorption of drug from the surface of niosomes. After the initial burst release, a constant AA release was observed during 360 min which was due to the diffusion of AA from lipid bilayer [64].

### 3.5. Fabrication of DMN

**Figure 6:**
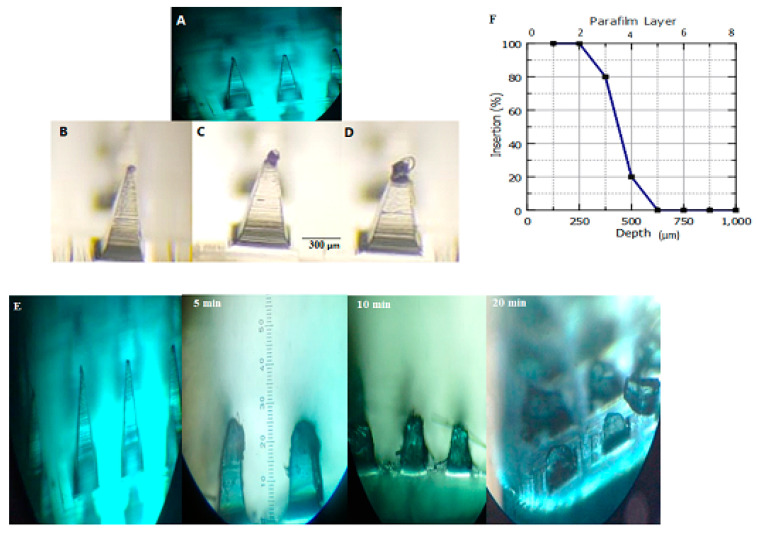
Polarizer photographs representing (**A**) morphology of MNs, (**B**) a decrease in the height of M4 after applying different pressures of 200 g, (**C**) 500 g, and (**D**) 1000 g, (**E**) Morphological changes of the needles after insertion into the rat skin for M4 at 5, 10, and 20 min. (**F**) Insertion depth and number of Parafilm M^®^ layers perforated during AA and CAFF-MN application.

In the study, PVP with a molecular weight of 40KDa was used as a matrix material to produce DMN. The aim was to evaluate the suitability and feasibility of various polymers for DMN fabrication. The selection of S4 and S7 as the drugs to be loaded into polymeric MNs was based on the characterization results, which showed they had the highest drug entrapment and release efficiencies. After optimizing the niosomes, DMN were prepared by combining the PVP polymer solution with the niosomal formulations. Methylene blue was loaded into DMNs for ease of visualization. The formed needles can be seen in Figure 6A. In the study, PVP with a molecular weight of 40 KDa was utilized as the matrix material for the production of DMN. This material is considered as a biodegradable and biocompatible [65]. Research has shown that the kidney can effectively remove PVP with a low molecular weight [9]. MNs array with 250 μm × 600 μm (width and height) based on a pyramidal MN master template was used to prepare MNs. The needles morphology was square pyramidal shape, because of their narrow aspect ratio, pyramidal MNs demonstrated more mechanical strength than conical MNs, according to previous studies [66]. The best formulations were fabricated by combining 60% PVP with a co-polymer of 5% PEG 400 and 60% PVP with a co-polymer of 7% PEG 400 exhibit the best mechanical properties. A previous study demonstrated that the combination of hydrophilic polymers in the MNs formulations resulted in the formation of MNs with better mechanical properties compared to using a single polymer. This combination also led to a faster dissolution rate [67]. Polymeric MNs are a promising technology with the advantage of the successful dermal release of a wide variety of drugs [42]. DMNs dissolve completely upon insertion into the skin and have high biocompatibility without leaving any sharps or biohazardous materials [68]. Additionally, DMNs require only one step-application [69]. When MNs is applied to the surface of the skin, it creates aqueous conduits with a number of small pores through where the nanoparticles can reach the deeper layers of the skin [70].

### 3.6. Mechanical Characterization of DMN

MNs must be mechanically robust to withstand handling and skin insertion [71]. When the weight pressed reached 1000 g. All MNs formulations showed less than 10% reduction in the height as summarized in Table 5. Therefore, DMN fabricated from PVP and PEG 400 showed good mechanical strength. Figure 6B–D shows a decrease in the height of DMN with increase in the forces applied. M1, M2, M3 and M4 were visualized after testing using a polarizer microscope and measuring the length of MNs. 

### 3.7. DMNs Dissolution in Skin

The study showed that DMN, prepared using PVP polymer solution combined with niosomal formulations, was applied to rat skin for various time intervals (5 min, 10 min, and 20 min). After 20 min, the MNs fully dissolved as shown in Figure 6E. The results indicated that higher content of PEG 400 in PVP facilitated the faster dissolution of MNs after piercing, making it important for cosmetic applications. Previous studies have also reported that increasing the content of PVP in MNs led to a faster dissolution rate in the skin and improved needle penetration ability [72].

### 3.8. Microneedles Insertion Studies

Figure 6F illustrates the ability of DMN to penetrate three to four layers of Parafilm M^®^. Given that each layer has an approximate thickness of 126 µm, it can be deduced that the DMN can penetrate depths ranging from 378 to 500 µm. This confirms that the needles are capable of reaching the SC and delivering the cargo effectively into the dermis layer. The results show that the fabricated DMNs are sturdy enough to penetrate the skin and quickly dissolve, ensuring efficient drug delivery.

### 3.9. Drug Content and Content Uniformity

The uniformity of the active ingredients within the drug delivery system (DMN) was evaluated by using two types of niosomal formulations (S4 and S7). The results, shown in Table 6, indicate that the average recovery rate of AA and CAFF from all four formulations of DMN was approximately 99% and 98%, respectively. No significant difference (*p* > 0.005) was observed in the recovery rate of AA and CAFF across all MNs formulations.

### 3.10. Stability Study

After two months, there was no alteration in the appearance of MNs and the moisture content did not vary significantly (*p* > 0.05) in M1, M2, M3, and M4. The recovery % and moisture content of DMN after one and two months for AA and CAFF are shown in Table 7. There was no significant difference (*p* > 0.05) in the recovery of AA and CAFF from all MNs after two months. The recovery % of AA and CAFF were 98.12 ± 0.45%, 97.87 ± 0.88% for M1, 98.64 ± 0.66%, 98.55 ± 1.40% for M2, 98.17 ± 0.75%, 97.52 ± 0.85% for M3, and 99.25 ± 0.58%, 98.76 ± 1.30% for M4 after two months, respectively. These findings indicate that both AA and CAFF niosomes were stable in terms of moisture content and drug content in all MNs formulations. Therefore, niosomes were proven to be an effective method for encapsulating hydrophilic and unstable compounds uniformly in polymeric matrices for the preparation of DMN.

### 3.11. Ex Vivo Permeation Study

**Figure 7:**
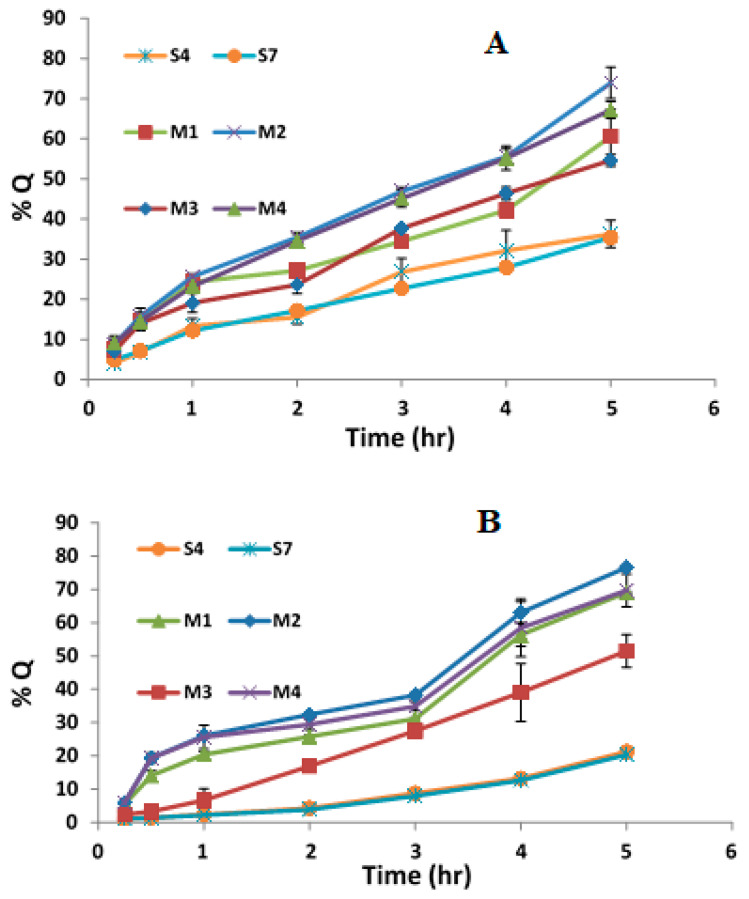
The permeation profile of (**A**) CAFF and (**B**) AA permeated across rat skin following the application of MNs and control (S4 and S7).

Table 8 presents the cumulative amount (Q), steady-state flux (Jss), and permeability of AA and CAFF permeated across rat skin after applying MNs loaded with AA and CAFF niosomes. Figure 7A,B show the permeation profile. After a 5 h period, the cumulative amount of AA and CAFF permeated from M1 was 616.891, 351.852 g/cm^2^, respectively, which represents 60.55%, 69.08% of AA and CAFF, respectively. M2 delivered 753.361, 390.061 µg/cm^2^, equating to 73.95%, 76.58% of AA and CAFF, respectively. M3 delivered 649.895, 306.125 µg/cm^2^, representing 54.59%, 51.5% of AA and CAFF, and M4 delivered 798.589, 414.205 µg/cm^2^, equating to 67.19%, 69.70% of AA and CAFF respectively, as shown in Table 8. The cumulative amount (Q) of AA and CAFF was in the following order: M4 (containing 7% PEG 400) > M2 (containing 7% PEG 400) > M3 (containing 5% PEG 400) > M1 (containing 5% PEG 400). Jss for different formulations was in the order: M4 (containing 7% PEG 400) > M2 (containing 7% PEG 400) > M1 (containing 5% PEG 400) > M3 (containing 5% PEG 400). The results indicate that using PVP and 7% PEG 400 together enhances skin permeation compared to using PVP and 5% PEG 400, which is in line with previous studies [73,74]. This improvement is attributed to the strong hydrophilic nature of PEG 400, which increases the solubilization of the drug and enhances partition to the aqueous phase [49].

Ex vivo skin permeation experiments revealed that the %Q observed using MNs loaded niosomes (passive and active method) was greater than %Q of AA and CAFF that permeated through S4 and S7 niosomes (passive method only). Since %Q of AA through S4 and S7 were 36.24 ± 3.43%, 35.35 ± 1.02%, respectively and %Q of CAFF through S4 and S7 were 21.24 ± 0.58%, 20.30 ± 0.72%, respectively as shown in Figure 7. The results of this study confirmed the fact that the encapsulation of drug in niosomes based DMN will facilitate the penetration of hydrophilic molecules AA and CAFF across *Stratum corneum* and improved the stability of AA since the flux of AA and CAFF through rat skin was enhanced more than 1.5-fold in comparison with niosomes alone. Many previous studies have confirmed that polymeric MNs could be utilized as an efficient tool for improving the penetration rate and delivery efficiency of an encapsulated drug [75,76].

### 3.12. Skin Deposition

The results of the ex vivo skin deposition analysis showed that the concentration of AA and CAFF in the full-thickness rat skin was significantly higher (*p* < 0.005) for the M3 formulation compared to other DMN formulations (M1, M2, and M4). The amount of AA deposited in the skin after 5 h was 941.08 ± 4.26, 30.40 ± 3.22, 43.70 ± 2.83, and 36.99 ± 3.69 from M1, M2, M3, and M4, respectively, as seen in Table 9. Similarly, the deposited amount of CAFF was 36.62 ± 3.26, 31.81 ± 3.26, 49.03 ± 6.95, and 30.64 ± 3.78 from M1, M2, M3, and M4, respectively. These findings support previous research by Permana, McCrudden, and Donnelly, who found that MNs loaded with nanocarrier systems significantly enhance drug retention times in the dermis [77]. There have been several studies investigating the preparation of ascorbic acid and caffeine as separate microneedle formulations. However, to the best of our knowledge, there has been no study exploring the incorporation of both compounds in a single microneedle formulation. Kim and colleagues [78] conducted a study where they developed a dissolving microneedle patch containing ascorbic acid 2-glucoside (AA2G) encapsulated in a needle-shaped hyaluronic acid (HA) backbone. To ensure product sterility, the MN patch was sterilized using electron beam (e-beam) and gamma ray (γ-ray) irradiation. The delivery of AA2G was found to increase and become saturated over 12 h and was completely delivered after 24 h. Chandran et al. [79] conducted a study to investigate the use of hydrogel microneedles (MN) as a permeation enhancer for transdermal delivery of caffeine. Additionally, the study found that hydrogel MNs fabricated with 3% *w*/*w* NaHCO3, and high MW of copolymer exhibited optimal physical and swelling properties for enhanced transdermal delivery. The results showed that approximately 2.9 mg of caffeine was delivered within 24 h.

### 3.13. Drug Release Kinetics

The drug release of AA and CAFF from M3 were perfectly following first order drug release model as the drug release profile of AA and CAFF are closest to trend line or regression line and there are the highest value of coefficient of correlation (R^2^ = 0.97), (R^2^ = 0.96), respectively as shown in Table 10. To understand the dissolution mechanisms from M3, the released data were fitted also using Korsmeyer-Peppas model. Anomalous transport is characterized by a release exponent value, n, that is between 0.5 and 1.0, indicating that the drug release is controlled by both the drug diffusion and other mechanisms [80,81]. The release exponent was found to be 0.96, and 0.99 respectively for AA and CAFF which implies that the drug release from the system follow anomalous (non-Fickian transport). Where both drug diffusion from niosomes structure and dissolution of the polymer controlled the release of AA and CAFF [82].

### 3.14. MTT Cytotoxicity Assay

Reactive oxygen species (ROS) are highly reactive molecules that play a crucial role in the development of cancer. Increased levels of ROS have been observed in various types of cancer cells and contribute to oxidative stress, leading to DNA damage and mutations [83]. However, the elevation of ROS levels beyond a cytotoxic limit leads to the death of cancer cells [84]. Furthermore, cancer cells have the ability to counteract high levels of ROS through the production of antioxidants and alteration of cellular metabolism.

The role of antioxidants in preventing oxidative damage to lipids and other macromolecules and preventing cancer formation has been well-established in the literature. Ascorbic acid (AA) and caffeine (CAFF) have been shown to have antioxidant properties [85,86].

**Figure 8:**
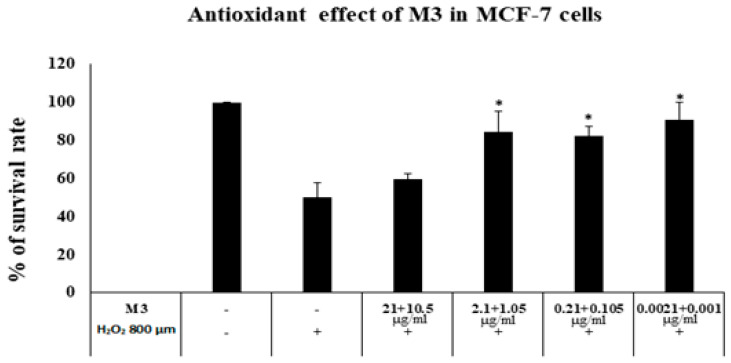
Antioxidant effect of formula M3 (AA + CAFF) on H_2_O_2_ induced oxidative stress in MCF-7 cells (Means + S.D.). * *p* < 0.05 compared with H_2_O_2_ only treated group.

Therefore, our objective in this study was to validate the well-known antioxidant properties of AA and CAFF in our novel niosomal formulation, referred to as M3, by testing its ability to prevent H_2_O_2_ induced cell damage and apoptosis in MCF-7 cells. When cells were treated with M3 for 48 h at concentration of 2.1 and 1.05 μg/mL for AA and CAFF respectively, a significant increase in cell viability (84.6%) was observed after H_2_O_2_ treatment, as compared to cells only treated with H_2_O_2_ (50%) (as shown in Figure 8). This result was only observed at concentrations below 2.1 and 1.05 μg/mL for AA and CAFF respectively, demonstrating the antioxidant properties of M3 and its capability to shield MCF-7 cells from H_2_O_2_ induced damage and apoptosis. While the use of M3 at these concentrations could be exploited to prevent cancer development, it is important to carefully evaluate the use of such formulations in cancer patients undergoing ROS-based treatments to ensure the complete effectiveness of such treatments.

**Figure 9:**
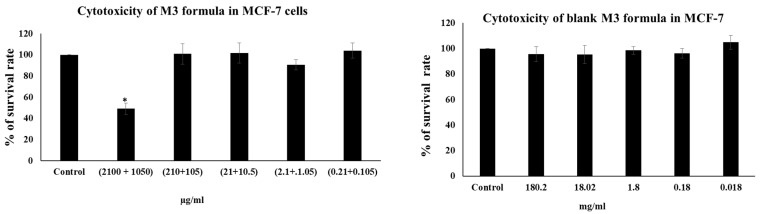
The cytotoxicity of M3 and blank M3 was evaluated by MTT assay (Means + S.D). * *p* < 0.05 compared with control.

Furthermore, in recent years, an increasing number of studies have reported the ability of millimolar concentrations of pharmacological AA to kill cancer cells in vitro and reduce tumor growth in vivo, including in the case of breast cancer [87,88]. Moreover, research has shown that high doses of caffeine are cytotoxic to MCF-7 cells in vitro at a concentration of 5 mM [89]. Therefore, to validate the anticancer properties of M3 formulation, we treated the MCF-7 cells with different serial dilutions. Only the highest concentration of M3 of (210 µg/mL) and CAFF (105 µg/mL), but not the blank formulation, inhibited the proliferation of MCF-7 cells by 50.9% (*p* < 0.05) (as shown in Figure 9). The mechanism behind the cytotoxic effect of AA and caffeine in cancer has been reported to involve the induction of H_2_O_2_. Readers are referred to review articles for a more in-depth examination of the mechanisms of AA and CAFF-induced cytotoxicity [90,91,92].

Based on these findings, we can confirm that AA and CAFF in our novel niosomal formulation (M3) possess antioxidant properties that can prevent H_2_O_2_ induced cell damage and apoptosis in MCF-7 cells at concentrations below 2.1 for AA and 1.05 for CAFF µg/mL. Additionally, M3 showed anticancer effects at concentrations of 210 µg/mL and 105 µg/mL for AA and CAFF, respectively. 

## 4. Conclusions

This is the first study to successfully optimize and incorporate a niosome containing AA and CAFF into a DMN system. The niosomes were uniform and without aggregation and the best encapsulation was achieved using a 1:1 ratio of surfactant to Chol. Loading these niosomes into DMN significantly improved the drug’s delivery to the skin. The skin deposition analysis showed that M3 had higher AA and CAFF deposition in rat skin than other formulations. The release profile of M3 was analyzed and determined to follow first-order kinetics. In addition, the results of our study suggest that the use of M3 as a cancer treatment is concentration dependent. It can be used at lower concentrations as a supplement to protect against antiaging or cancer and at high concentrations to kill cancer cells. Further studies are needed to better understand the optimal concentration and administration of M3 as a cancer treatment and to determine its efficacy in various types of cancer. Further investigation should also be conducted to determine the potential toxic effects of M3, especially at high concentrations, to ensure its safety for clinical use. In conclusion, using MN technology in combination with niosomal carriers could be a promising delivery system for hydrophilic drugs applied to the skin.

## Figures and Tables

**Table 1:** The composition of AA and CAFF niosomal nanoparticle formulations.

Code	Span 60	Tween 60	Tween 80	Chol	DCP	CAFF	AA	HLB Value
	(mg)	(mg)	(mg)	(mg)	(mg)	(mg)	(mg)	
S1	150	-	-	150	2	50	100	4.7
S2	300	-	-	150	2	50	100	4.7
S3	150	-	-	75	2	50	100	4.7
S4	125	25	-	150	2	50	100	6.4
S5	250	50	-	150	2	50	100	6.4
S6	125	25	-	75	2	50	100	6.4
S7	120	-	30	150	2	50	100	6.8
S8	240	-	60	150	2	50	100	6.8
S9	120	-	30	75	2	50	100	6.8

**Table 2:** Composition of AA and CAFF niosomal nanoparticles in MNs.

Code	PVP	PEG 400	AA and CAFF Niosomes
M1	95	5	0.3 mL of S4 niosomes
M2	95	5	0.3 mL of S7 niosomes
M3	93	7	0.3 mL of S4 niosomes
M4	93	7	0.3 mL of S7 niosomes

**Table 3:** The PS, PDI, ZP, and EE% of prepared niosomal formulation. Results are represented by mean ± SD (n = 3).

Code	PS (nm)	PDI	ZP (mV)	EE (%)	
				CAFF	AA
S1	150.70 ± 10.10	0.005 ± 0.00	−27.53 ± 0.54	35.83 ± 1.20	39.68 ± 1.30
S2	170.00 ± 3.70	0.031 ± 0.00	−25.40 ± 0.52	15.16 ± 0.99	10.88 ± 1.51
S3	130.60 ± 7.50	0.255 ± 0.03	−30.30 ± 0.80	16.37 ± 0.11	13.78 ± 1.82
S4	411.20 ± 4.51	0.027 ± 0.01	−41.13 ± 7.41	59.71 ± 0.09	61.98 ± 0.19
S5	302.90 ± 1.50	0.228 ± 0.00	−50.20 ± 3.74	42.76 ± 0.25	35.12 ± 1.72
S6	391.50 ± 1.10	0.213 ± 0.02	−48.30 ± 2.10	45.87 ± 0.51	36.42 ± 1.26
S7	369.50 ± 3.21	0.204 ± 0.03	−40.20 ± 5.61	67.07 ± 0.30	70.89 ± 0.42
S8	280.10 ± 0.60	0.256 ± 0.00	−40.70 ± 0.77	37.97 ± 0.69	42.52 ± 0.49
S9	386.50 ± 2.34	0.005 ± 0.00	−41.76 ± 0.64	49.90 ± 0.15	43.13 ± 0.25

**Table 4:** The short-term stability results of S4, and S7 at 4 °C for 2 months. PS, PDI, ZP and %EE studied as stability parameters. Results are represented by mean ± SD (n = 3).

Code		PS (nm)	PDI	ZP (mv)	%EE	
					AA	CAFF
S4	Freshly prepared	411.20 ± 4.51	0.02 ± 0.01	−41.13 ± 7.41	59.71 ± 0.09	61.98 ± 0.19
	One month	415.50 ± 6.50	0.25 ± 0.01	−36.5 ± 7.66	61.32 ± 2.02	58.99 ± 2.04
	Two months	410.30 ± 7.50	0.221 ± 0.02	−39.3 ± 6.30	59.46 ± 0.44	56.09 ± 0.34
S7	Freshly prepared	369.50 ± 3.21	0.20 ± 0.03	−40.20 ± 5.61	67.07 ± 0.30	70.89 ± 0.42
	One month	374.30 ± 5.90	0.11 ± 0.01	−33.8 ± 0.53	69.77 ± 0.02	67.80 ± 0.01
	Two months	363.00 ± 4.50	0.14 ± 0.00	−36.7 ± 0.66	69.76 ± 0.40	66.96 ± 0.14

**Table 5:** The reduction in height (μm) of (M1, M2, M3 and M4 tested as a function of forces of 200, 500 and 1000 g per array (Mean ± SD, n = 3).

Formulation	Forces Applied per Array			
	Control	200 gm	500 gm	1000 gm
M1	595 ± 10	580 ± 17	575 ± 30	565 ± 17
M2	600 ± 00	590 ± 00	570 ± 17	560 ± 30
M3	593 ± 05	585 ± 17	580 ± 17	570 ± 17
M4	600 ± 00	590 ± 00	570 ± 34	560 ± 30

**Table 6:** The percentage recovery of AA and CAFF from DMN ^a^.

Formulation	% Recovery	
	AA	CAFF
M1	99.33 ± 1.11	98.55 ± 0.17
M2	99.66 ± 0.47	99.19 ± 0.15
M3	98.72 ± 0.79	98.03 ± 0.40
M4	99.67 ± 0.41	99.25 ± 0.50

^a^ Data are presented as Mean ± SD, n = 3.

**Table 7:** Moisture content percentage and recovery percentage for M1, M2, M3 and M4 after one- and two-months ^a^.

Formulations	%Moisture Content		%Recovery			
	One Month	Two Months ^b^	One Month		Two Months ^b^	
			AA	CAFF	AA	CAFF
M1	4.35 ± 0.12	5.17 ± 0.16	98.25 ± 1.05	98.25 ± 0.29	98.12 ± 0.45	97.87 ± 0.88
M2	4.76 ± 0.13	5.98 ± 0.15	98.71 ± 0.25	98.97 ± 0.94	98.64 ± 0.66	98.55 ± 1.40
M3	3.56 ± 0.11	3.71 ± 0.05	98.21 ± 0.25	97.89 ± 0.67	98.17 ± 0.75	97.52 ± 0.85
M4	2.22 ± 0.10	2.54 ± 0.09	99.14 ± 0.23	99.05 ± 1.20	99.25 ± 0.58	98.76 ± 1.30

^a^ Data are presented as Mean ± SD, n = 3. ^b^ There was no significant difference (*p* > 0.05) in the moisture content and recovery of AA and CAFF from all MNs after two months.

**Table 8:** Drug permeation parameters from different formulation at 37 °C ^a^.

Code		Q%	Q/A (µg/cm^2^)	Jss (µg/cm^2^/h)	P × 10^−2^ (cm/h)
M1	AA	60.55 ± 6.82	616.8 ± 69.5	110.1 ± 4.2	6.1 ± 0.3
	CAFF	69.08 ± 0.33	351.8 ± 85.2	78.9 ± 3.6	8.8 ± 0.2
M2	AA	73.95 ± 3.93	753.3 ± 40.1	126.2 ± 6.5	7.0 ± 0.2
	CAFF	76.58 ± 0.39	390.0 ± 2.0	80.1 ± 4.3	8.9 ± 0.02
M3	AA ^b^	54.59 ± 1.43	649.8 ± 17.7	100.8 ± 6.3	4.8 ± 0.04
	CAFF ^b^	51.51 ± 7.90	306.1 ± 47.1	71.3 ± 3.6	6.8 ± 0.02
M4	AA	67.19 ± 2.08	798.5 ± 19.2	128.4 ± 8.4	6.1 ± 0.07
	CAFF	69.70 ± 4.92	414.2 ± 29.2	85.7 ± 5.3	8.2 ± 0.02

^a^ Data are presented as Mean ± SD, n = 4. ^b^ The permeation parameters of M3 were significantly lower (*p* < 0.05) compared to other MN formulations.

**Table 9:** %AA and CAFF remained in skin from different formulation ^a^.

	% Deposited in Skin	
	CAFF	AA
M1	36.62 ± 3.26	41.08 ± 4.26
M2	31.81 ± 3.26	30.40 ± 3.22
M3	49.03 ± 6.95 ^b^	43.70 ± 2.83 ^b^
M4	30.64 ± 3.78	36.99 ± 3.69

^a^ Data are presented as Mean ± SD, n = 3. ^b^ The concentration of AA and CAFF in the full-thickness rat skin was significantly higher (*p* < 0.005) for the M3 formulation compared to other MN formulations.

**Table 10:** Kinetics governing the release of AA and CAFF through M3.

API	First Order Model		Korsmeyer-Peppas Model		
	R^2^	K	R^2^	K	n
AA	0.9723	0.003	0.9548	0.231	0.962
CAFF	0.9659	0.002	0.9851	0.172	0.994

## Data Availability

Not applicable.
